# Visfatin promotes intervertebral disc degeneration by inducing IL-6 expression through the ERK/JNK/p38 signalling pathways

**DOI:** 10.1080/21623945.2021.1910155

**Published:** 2021-04-14

**Authors:** Haitao Cui, Xianfa Du, Caijun Liu, Shunlun Chen, Haowen Cui, Hui Liu, Jianru Wang, Zhaomin Zheng

**Affiliations:** a Department of Spine Surgery, The 1st Affiliated Hospital of Sun Yat-sen University, Guangzhou, Guangdong, PR China; b The Third Affiliated Hospital of Guangzhou, University of Traditional Chinese Medicine, Guangzhou, Guangdong, PR China; c Pain Research Center, Sun Yat-sen University, Guangzhou, PR China

**Keywords:** Adipocytokine, visfatin, il-6, aggrecan, type ii collagen, ivdd

## Abstract

Visfatin reportedly induces the expression of proinflammatory cytokines. Severe grades of intervertebral disc disease (IVDD) exhibit higher expression of visfatin than mild ones. However, the direct relationship between visfatin and IVDD remains to be elucidated. This study aimed to clarify whether stimulation of visfatin in IVDD is mediated by IL-6. To investigate the role of visfatin in IVDD, a rat model of anterior disc puncture was established by injecting visfatin or PBS using a 27-gauge needle. Results revealed an obvious aggravation of the histological morphology of IVDD in the visfatin group. On treating human NP cellswith visfatin, the levels of collagenII and aggrecan decreased and those of matrix metallopeptidase 3 and IL-6 gradually increased. A rapid increase in ERK, JNK, and p38 phosphorylation was also noted after visfatin treatment. Compared to those treated with visfatin alone, NP cells pretreated with ERK1/2, JNK, and p38 inhibitors or siRNA targeting p38, ERK, and JNK exhibited a significant suppression of IL-6. Our data represent the first evidence that visfatin promotes IL-6 expression in NP cells via the JNK/ERK/p38-MAPK signalling pathways. Further, our findings suggest epidural fat and visfatin as potential therapeutic targets for controlling IVDD-associated inflammation.

## Introduction

Low back pain (LBP), one of the most common health problems worldwide, places an enormous global burden on public health and social economy [1,2]. Intervertebral disc disease (IVDD) is a multifactorial disease that plays an important role in LBP. The intervertebral disc (IVD) is composed of distinct sub-structures: the centrally situated and gelatinous nucleus pulposus (NP) and the fibrocartilaginous annulus fibrosus on the radial periphery. NP consists of NP cells and abundant extracellular matrix (ECM), which is rich in proteoglycans, primarily type II collagen and aggrecan [3,4]. Reduced ECM and an imbalance between anabolism and catabolism characterize IVDD [5]. In addition, recent studies have reported that high levels of proinflammatory factors also play an important role in inducing IVDD [6,7].

Adipose tissue, commonly called fat, not only provides sufficient cushion and energy to the body, but also serves as an endocrine organ. Proteins secreted by adipose tissue are actively involved in the regulation of neuroendocrine, autonomic, and immune functions and in the maintenance of energy homoeostasis [8–10]. Adipose tissues are not only an active tissue, there are fat pads also involved in the development of some diseases. Belluzzi E and his colleagues found that infrapatellar fat in osteoarthritis patients were more inflamed and vascularized compared to infrapatellar fat from patients undergoing anterior cruciate ligament [11]. Adipose tissue also play a role in the pathophysiology of patients with heart failure. Heart failure patients had more epicardial fat compared to controls [12]. Epidural fat provides sufficient cushioning for the pulsatile movements of the dural sac, protects nerve structures, and facilitates movement of the dural sac over the periosteum of the spinal column during flexion and extension. As an endocrine organ, epidural fat can also secrete adipocytokines that act locally or reach distant tissues via systemic circulation. Leptin plays an important role in IVDD pathology. It initiates degradative and inflammatory cascades in disc cells, enhances disc cell proliferation, and mediates ECM degradation [13–15]. Resistin can augment the expression of chemokine CC motif ligand 4 (CCL4) by directly binding to the toll-like receptor 4 (TLR4) on degenerated human NP tissues [16].

Nicotinamide phosphoribosyltransferase (NAMPT), also called pre-B cell colony-enhancing factor (PBEF) or visfatin, is an adipocytokine that promotes the production of interleukin 6 (IL-6) and tumour necrosis factor alpha (TNF-α) in human synovial fibroblasts [17]. APO866, an inhibitor of NAMPT, protects NP cells and inhibits IL-1β-induced ECM degeneration by autophagy [18]. However, the direct relationship between visfatin and the development of IVDD remains to be elucidated.

IL-6 is a classical cytokine that maintains homoeostasis [19] and serves as a soluble mediator with pleiotropic effect on immune response, inflammation, and haematopoiesis [20]. IL-6 can potentiate the catabolic actions of IL-1 and TNF-α in NP cells [21]. IL-6 could also significantly elevate the levels of prostaglandin E2 (PGE-2) and matrix metalloproteinase 13 (MMP-13) and decrease proteoglycan synthesis in NP cells [22]. Furthermore, IL-6-mediated expression of TNF-α in the dorsal root ganglion (DRG) may contribute to the development of allodynia and hyperalgesia [23,24]. Further support regarding the contribution of IL-6 to sciatic pain was obtained from the discovery that genetic variations in IL-6 have a relationship with internal disc disruption (IDD)-related radiculopathy [25]. Notably, IL-6 has a significant relationship with IVDD and LBP.

This study investigated whether upregulation of visfatin induces IL-6 expression and reduces that of type II collagen and aggrecan in NP cells. Further, the role of epidural fat of the spinal column in the pathology of IVDD was also evaluated. Our results showed that the expression of visfatin and IL-6 was significantly decreased in epidural adipose tissues compared with subcutaneous adipose tissues in the patients with lumbar spinal stenosis or disc herniation. Moreover, visfatin could aggravate IVDD by reducing the expression of type II collagen and aggrecan and increasing the expression of IL-6.

## Materials and methods

### Human tissue collection

From October 2018 to December 2019, subcutaneous and epidural adipose tissues were obtained from nine patients (four males and five females range 25–76 years) during surgery (lumbar spinal stenosis: six cases; lumbar disc herniation: three cases) at the First Affiliated Hospital of Sun Yat-sen University, Guangzhou, China. None of the patients had systemic diseases, such as rheumatoid arthritis or diabetes mellitus. Disc samples were collected from three patients (two males and one female range 19–26 years) who underwent surgical interventions (Lumbar spondylolysis: three cases) at the First Affiliated Hospital of Sun Yat-sen University, Guangzhou, China. The information of human samples from 12 patients is listed in (Table 1). Each patient provided informed consent prior to participation in the study, which was approved by the Ethics Committee of our institution (First Affiliated Hospital of Sun Yat-sen University).

**Table 1.** Information of human samples from 12 patients

Sex	Age	Body Mass Index(BMI)	Sample
Female	64	27.3	subcutaneous and epidural adipose tissues
Male	63	18.5	subcutaneous and epidural adipose tissues
Female	25	20.5	subcutaneous and epidural adipose tissues
Male	76	28.1	subcutaneous and epidural adipose tissues
Female	53	25.3	subcutaneous and epidural adipose tissues
Female	33	19.5	subcutaneous and epidural adipose tissues
Female	69	23.4	subcutaneous and epidural adipose tissues
Male	59	23.3	subcutaneous and epidural adipose tissues
Male	29	21.6	subcutaneous and epidural adipose tissues
Male	19	20.8	intervertebral disc
Female	23	17.5	intervertebral disc
Male	26	20.6	intervertebral disc

### Diﬀerentially Expressed Genes (DEGs) screening

Briefly, mRNA from subcutaneous and epidural adipose tissues was isolated from total RNA with NEBNext® Poly(A) mRNA Magnetic Isolation Module (New England Biolabs). Alternatively, rRNA is removed from the total RNA with a RiboZero Magnetic Gold Kit (Human) (Epicentre, an Illumina Company). The enriched mRNA or rRNA depleted RNA was used for RNA-seq library preparation using KAPA Stranded RNA-Seq Library Prep Kit (Illumina). DEGs analysis was conducted using a TruSeq SR Cluster Kit v3-cBot-HS (#GD-401-3001, Illumina). Sequencing was carried out using the Illumina HiSeq 4000 according to the manufacturer’s instructions. Sequencing was carried out by running 150 cycles. Principle Component Analysis (PCA) and correlation analysis were based on gene expression level, Hierarchical Clustering, scatter plots and volcano plots were performed with the differentially expressed genes in R, Python or shell environment for statistical computing and graphics. To provide a comprehensive landscape of the inflammatory cytokines and adipocytokines involved in IVDD and produced in the epidural adipose tissue compared to the subcutaneous adipose tissue, representative DEGs of the cytokines and adipocytokines are shown in the heatmap.

### Rat model of IVDD

All animal experiments were approved by the Animal Care and Use Committee of the sixth Affiliated Hospital of Sun Yat-sen University. The research procedures were in accordance with the ‘Guidelines of Experiments on Animals’. Fifteen Sprague-Dawley rats weighing 250‒300 g and aged 9‒10 weeks were used in this study. The disc degeneration model described in our previous study, wherein we established a model of anterior disc puncture IVD degeneration, was used [26,27]. The rats were randomly divided into three groups: non-puncture group, phosphate-buffered saline (PBS) group (rats were punctured and injected with 2 µL of PBS), and visfatin group (rats were punctured and injected with 8 ng/µL of visfatin in 2 µL PBS), with five rats in each group. A 27-gauge needle was used for injection into the L4/5 IVD.

Magnetic resonance imaging was performed after 0, 2, and 4 weeks of initial annulus puncture. We used the Pfirrmann classification to assess the degree of IVDD [28]. The grade of IVDD was according to its structure, distinction of nucleus and anulus, signal intensity and height of intervertebral disc. (1 point = Grade I, 2 points = Grade II, 3 points = Grade III, 4 points = Grade IV, 5 points = Grade V).

Blood samples were obtained at the end of the experiment and centrifuged for 15 min at 3000 RPM. Than the rats were euthanized in a CO_2_ chamber and the L4/5 IVDs were excised along with the adjacent vertebrae.

### Immunohistochemistry and histopathological analysis

Histological examinations were performed using the injected IVDs obtained from the three groups at the indicated time points and adipose tissues from four patients we mentioned above. After the rats were euthanized, the injected IVDs were excised along with the adjacent vertebrae. The specimens were then decalcified, embedded in paraffin, and cut into 5-µm sections. Subsequently, the the sections were deparaffinised and rehydrated, followed by Safranin O/fast green or haematoxylin and eosin (H&E) staining or antigen retrieval with 0.01 mol·L^−1^ sodium citrate. The sections were blocked with 5% normal goat serum and 3% hydrogen peroxide. Then, the slides were incubated with the following primary antibodies: anti-IL-6 (1:50; Zen BioScience, 500,286), anti-type II collagen (1:200; Abcam, ab34712), anti-aggrecan (1:50; Abcam, ab3778), anti-monocyte chemotactic protein 1 (MCP-1) (1:200; Proteintech, 25,542-1-AP) and anti-vascular endothelial growth factor (VEGF) (1:200; Proteintech, 19,003-1-AP). The sections were incubated with a secondary antibody (1:200; Sevicebio, GB23303) (1:200; abcam,ab205718) and developed with 3,3′-Diaminobenzidine solution. Finally, the sections were observed and imaged; the percentages of IL-6^+^, type II collagen^+^, aggrecan^+^, MCP-1^+^ and VEGF^+^ cells in the samples were quantified using ImageJ software (National Institutes of Health, Bethesda, MD, USA). The IVDs histological staining scores were graded according to the criteria modified by Mao et al [29]. The histologic score was 5 for normal disc, 6–11 for moderately degenerated disc and 12–14 for severely degenerated disc. The adipose tissues histological staining scores were graded according to the criteria modified by Favero M et al [30].

### Isolation and culture of NP cells

NP cells were isolated from the collected human disc samples. The NP tissues were cut into pieces, treated with 0.1% collagenase and 10 U/ml hyaluronidase for 4–6 h separately. The partially digested tissue was incubated as an explant in Dulbecco’s modified Eagle’s medium (DMEM, Gibco, 8,120,376) and 10% foetal bovine serum (Gibco, 1,872,300) supplemented with antibiotics (Gbico, 15,140–122) in a humidified atmosphere containing 5% CO_2_ at 37°C. The method was reported earlier by Risbud et al [31].

### Real-time quantitative reverse transcription polymerase chain reaction (RT-qPCR)

Total RNA from NP cells was extracted using an RNA purification kit (Sangon, RN001) following the manufacturer’s protocols. A PCR master mix kit (Toyobo, 0910–012700) was used to remove the genomic DNA and to reverse-transcribe RNA into complementary DNA (cDNA). Custom-designed primers targeting mRNA encoding IL-6, type II collagen, and visfatin were synthesized for qPCR. PCR using primers and cDNA templates was performed with SYBR Green Mix (Roche, 47,138,600). Glyceraldehyde 3-phosphate dehydrogenase was used as an internal control. The relative mRNA expression levels were quantified using the 2-ΔΔCt method. All RT-qPCR experiments were carried out in triplicates. The primer sequences used are listed in (Table 2).

**Table 2.** Primer sequences (human)

Gene	Forward prime (5ʹ-3ʹ)	Reverse prime (5ʹ-3ʹ)
IL-6	ACTCACCTCTTCAGAACGAATTG	CCATCTTTGGAAGGTTCAGGTTG
Visfatin	GTGACTTAAGCAACGGAGCG	GGAGGATGTTGAACTCGGCT
Aggrecan	GCACAGCCACCACCTACAAAC	CTTTGGCATTCGGCGGACAA
Collagen II	ATGAGGGCGCGGTAGAGAC	TCACAGACACAGATCCGGCA
MMP3	GGTTCCGCCTGTCTCAAGAT	AGGGATTTGCGCCAAAAGTG
ERK1/2	TACACCAACCTCTCGTACATCG	CATGTCTGAAGCGCAGTAAGATT
JNK	TGTGTGGAATCAAGCACCTTC	AGGCGTCATCATAAAACTCGTTC
p38	CCAGGGGCTGAGCTTTTGAA	TCGGCCACTGGTTCATCATC
GAPDH	GACAGTCAGCCGCATCTTCTT	AATCCGTTGACTCCGACCTTC

### Western blotting

Total protein extracted from NP cells using a radioimmunoprecipitation assay buffer (Sigma–Aldrich, 223,410) was quantified using the bicinchoninic acid assay (Thermo Fisher Scientific, va294778). The total cellular proteins were then resolved with 10‒12% sodium dodecyl sulphate-polyacrylamide gel electrophoresis and transferred to polyvinylidene fluoride membranes (Millipore) via electroblotting. Thereafter, the membrane was blocked with 5% dry skimmed milk in tris-buffered saline with 0.1% Tween (TBST) and incubated overnight with the following primary antibodies at 4°C overnight: anti-IL-6 (1:1000; MultiSciences, 70-ab36529-100), anti-type II collagen (1:1000; Abcam, ab34712), anti-aggrecan (1:100; Abcam, ab3778), anti-MMP3 (1:1000; Abcam, ab52915), anti-ERK1/2 (1:1,000; CST, 4695S), anti-phospho-ERK1/2 (1:1,000; CST, 4370S), anti-JNK (1:1,000; CST, 9252S), anti-phospho-JNK (1:1,000; CST, 9255S), anti-p38 (1:1,000; CST, 8690S), anti-phospho-p38 (1:1,000; CST, 4511S), and anti-β-actin antibodies (1:5000; CST, 4970S). Post-incubation, the membranes were washed with TBST and incubated again with anti-rabbit (1:5000, Proteintech, SA00001-2) or anti-rat (1:5000, Proteintech, SA00001-1) horseradish peroxidase-conjugated secondary antibodies at room temperature for one hour. Finally, a multi-gauge densitometry system (Fujifilm, Tokyo, Japan) was used to quantify the results of this experiment.

### Enzyme-linked immunosorbent assay (ELISA)

NP cells were trypsinized, dispersed into single-cell suspensions and seeded in six-well plates. Each well contained 5 × 104 cells. Then the culture medium was added with visfatin (PeproTech, 96–130-09-25). The concentration of IL-6 in the cell supernatant was detected using an IL-6 ELISA Kit (MultiSciences, 70-EK106/2-96) and visfatin in the rats plasma collected from the rats blood samples was detected using an Visfatin ELISA Kit (Abcam, ab267799), according to the manufacturer’s instructions.

### MAPK inhibitors and pretreatment

One day before pretreatment, human NP cells were seeded in a six-well plate (5 × 10^6^ cells per well). Dimethyl Sulphoxide was used to dissolve ERK1/2 inhibitor (PD98059, CST, 9900S, 20 mM), JNK inhibitor (SP600125, CST, 8177S, 25 mM), or p38 inhibitor (SB203580, CST, 5633S, 20 mM). Then these inhibitors (1:1000) were transferred to the above six-well plate with 2 mL of DMEM. After 1 h of incubation, the culture medium was added with visfatin (PeproTech, 96–130-09-25). After one day, the cells were harvested for RNA and protein extraction.

### Small interfering RNA and transfection

Human ERK-, JNK-, and p38-targeting small-interfering RNAs (siRNAs) used in this study were constructed by RiboBio (Guangzhou, China). The sequences of the siRNAs are listed in (Table 3). One day before transfection, human NP cells were seeded in a six-well plate (5 × 10^6^ cells per well). Serum-free Opti-MEM (200 µL, Gbico, 2,174,121) was used to dissolve 5 µL of siRNA or 5 μL of Lipofectamine 3000 (Invitrogen, 2,262,699) individually. After mixing together and incubating for 15 min, the mixture was transferred to the above six-well plate with 2 mL of Opti-MEM. After 8 h of incubation, the culture medium was replaced with fresh complete medium. After two days, the cells were harvested for RNA extraction.

**Table 3.** siRNA sequences (human)

Gene	Sense (5ʹ-3ʹ)	Antisense (5ʹ-3ʹ)
ERK1/2 siRNA	CCGAAGCACCAUUCAAGUUTT	AACUUGAAUGGUGCUUCGGTT
JNK siRNA	CACCAAAGAUCCCUGACAATT	UUGUCAGGGAUCUUUGGUGTT
p38 siRNA	UGGUCUGUUGGACGUUUUUTT	AAAAACGUCCAACAGACCATT

### Statistical analysis

The experiments were performed in triplicates. The data are presented as mean ± standard deviation. A P-value< 0.05 was considered statistically significant. Student’s t-test and one-way of variance were used to analyse the differences between the groups with Shapiro-Wilk test for normality test. The result of Levene’s test was used to determine the post hoc testing strategy. If the result is significant, Dunnett’s T3 post hoc test for unequal variance was used, while LSD-t post hoc test was employed if there is no significant result. Statistical analysis was performed using SPSS 23.0 software (IBM, Armonk, NY, USA).

## Results

### Expression of visfatin and IL-6 was suppressed in human epidural fat compared to that in subcutaneous fat

**Figure 1.**
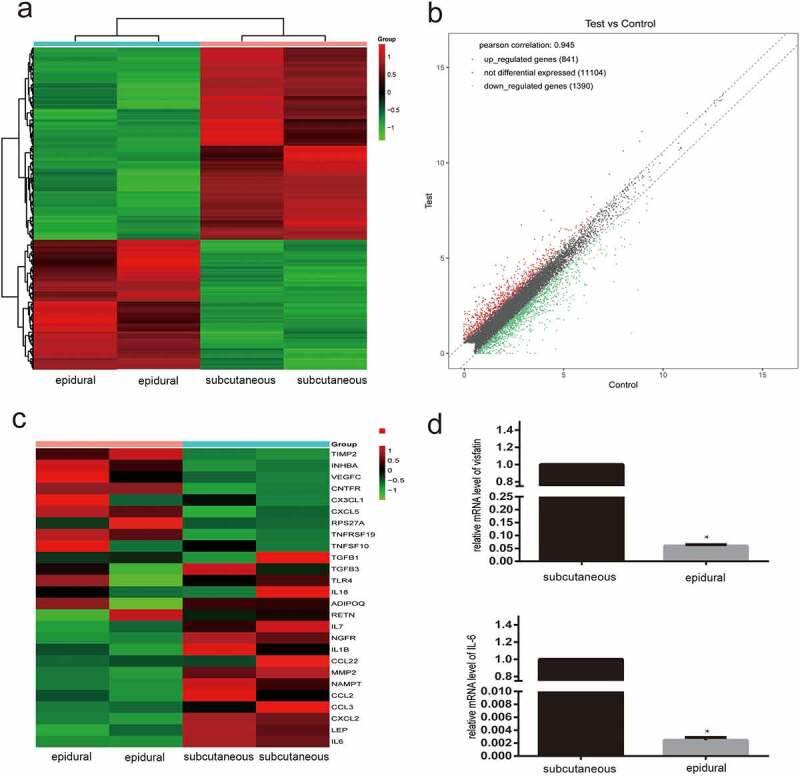
Diagrams of the differentially expressed genes (DEGs) in the epidural fat and subcutaneous fat. (a) Heatmap of the DEGs is shown for the RNA fraction. Data are displayed with different samples in the rows (two biological replicates for each group). Colour intensities represent gene upregulation (red) and downregulation (green). (b) Scatter plot of log-10 reads per kilobase per million mapped reads (RPKM) values representing each gene. (c) Heatmap of the DEGs of cytokines and adipocytokines involved in intervertebral disc disease. (d) Real-time quantitative reverse transcription polymerase chain reaction analysis of visfatin and IL-6 levels in epidural and subcutaneous fat. N = 3. *P < 0.001 versus subcutaneous

To investigate the differences in the expression of adipocytokines and cytokines in the subcutaneous and epidural adipose tissues of patients with lumbar spinal stenosis or lumbar disc herniation, differentially expressed genes (DEGs), with an overall false discovery rate ≤0.05 and logarithm of fold change value ≥±2, were identified by paired comparisons of the epidural and subcutaneous adipose tissues. Considering these results, representative heatmaps for total genes allowed us to visualize the expression pattern of the DEGs (Figure 1a). Differences in the gene expression profiles of the epidural and subcutaneous adipose tissues were observed in both the replicates. In addition, log-10 reads per kilobase per million mapped reads (RPKM) values for genes in epidural and subcutaneous adipose tissues illustrated in a scatter plot (Figure 1b) showed that the expression levels of the majority of the genes were similar in both epidural and subcutaneous adipose tissues (grey central blur), while only some genes were differentially expressed (red and green points for up-regulated and down-regulated DEGs, respectively). Considering all the analyses, we identified the DEGs.

To provide a comprehensive landscape of the inflammatory cytokines and adipocytokines involved in IVDD and produced in the epidural adipose tissue compared to the subcutaneous adipose tissue, representative DEGs of the cytokines and adipocytokines are shown in the heatmap (Figure 1c). Overall, differential expression of the cytokines and adipocytokines was greater in the epidural adipose tissue than in the subcutaneous adipose tissue. Representative cytokines included IL-6, leptin, CCL2, CCL3, CXCL2, and visfatin. A significant decrease in the expression of visfatin and IL-6 were noted in the epidural adipose. The downregulation was then verified by PCR in the epidural and subcutaneous adipose tissues (Figure 1d).

### Histological analysis of epidural fat compared to the subcutaneous one

**Figure 2.**
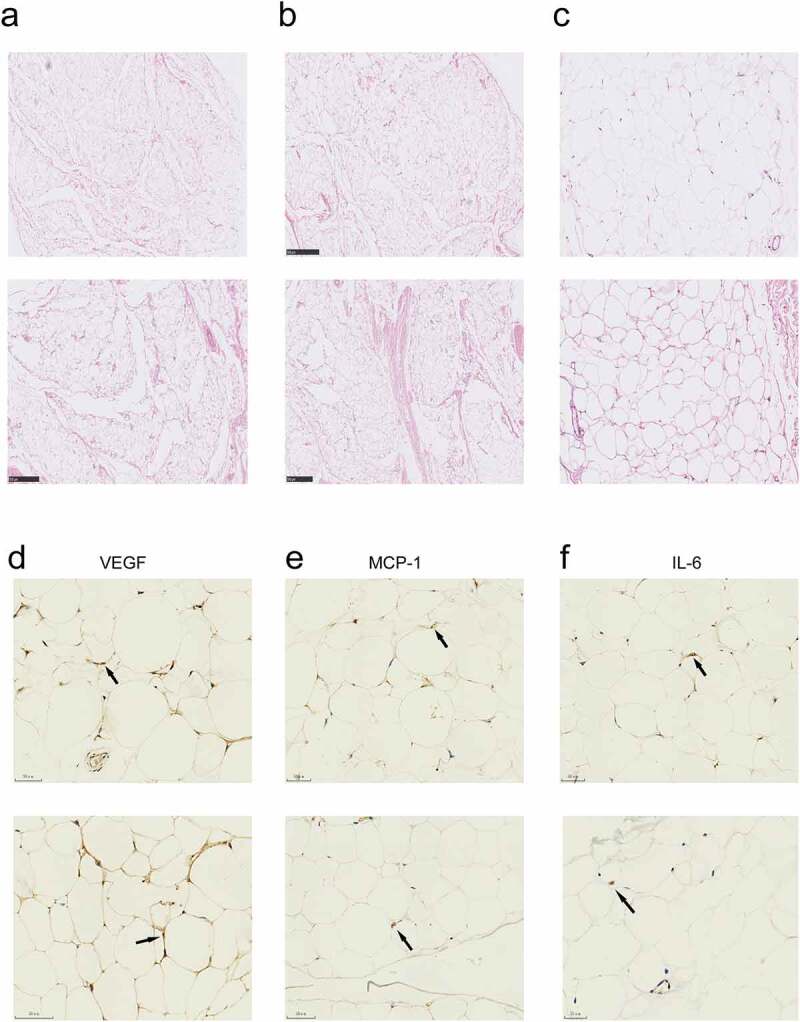
Histological characteristics of the epidural and subcutaneous fat. (a, b) Microscopic appearance showed the the mean diameter of adipose lobuli and the thickness of the interlobular septa were similar in the two groups . (c) There was no mononuclear infiltration in both groups. a-c, haematoxylin-eosin. Scale bars: (a, b), 500 μm; (c), 150 μm. (d) Vascularization evaluated by IHC with anti-VEGF of the epidural and subcutaneous fat. (e, f) MCP-1 and IL-6 immunostaining showed isolated roundish cells with positive cytoplasm in the both groups. Scale bars:(d, e, f), 50 μm

To investigate whether the state of epidural fat and subcutaneous one was rather different, epidural and subcutaneous fat samples collected from four patients underwent histologic and morphologic analysis. Then the H&E staining and Immunohistochemical (IHC) staining data were recorded. The fat histolopathologic and immunohistochemistry scoring system were showed in (Table 4). No significant differences were detected in the mean diameter of adipose lobuli, the thickness of the interlobular septa and the vascularity (evaluated both in HE-stained and anti-VEGF stained sections) of epidural fat compared with subcutaneous one (Figure 2a,b,c). Lymphocytic infiltration was not observed in both adipose tissues (c). The percentage of VEGF, MCP-1 and IL-6 isolated positive cells were observed in both adipose tissues(Figure 2d,e,f) .

**Table 4.** Fat histolopathologic and immunohistochemistry scoring system

AI histopathologic grading	Epidural AI	Subcutaneous AI	*P*-value
Lymphocytic infiltration, n (%)			ns
Grade 0	4 (100)	4 (100)	
Grade 1			
Grade 2			
Vascularity, in HE, number, mean (S.D.)	7.250 (1.109)	10.75 (2.548)	0.1157 (ns)
Thickness of the interlobular septa, mean (S.D.), mm	0.200 (0.015)	0.2487 (0.016)	0.0994 (ns)
Diameter of adipose lobuli, mean (S.D.), mm	1.293 (0.122)	1.237 (0.103)	0.7409 (ns)
IFP immunohistochemistry grading			
VEGF, n (%)			ns
Grade 1	4 (100)	4 (100)	
Grade 2	0 (0)	0 (0)	
Grade 3	0 (0)	0 (0)	
MCP-1, n (%)			ns
Grade 0	4 (100)	3 (75)	
Grade 1	0 (0)	1 (25)	
Grade 2	0 (0)		
IL-6, n (%)			ns
Grade 0	2 (50)	1 (25)	
Grade 1	2 (50)	3 (75)	
Grade 2	0 (0)	0 (0)

### Visfatin induces IVDD in vivo

**Figure 3.**
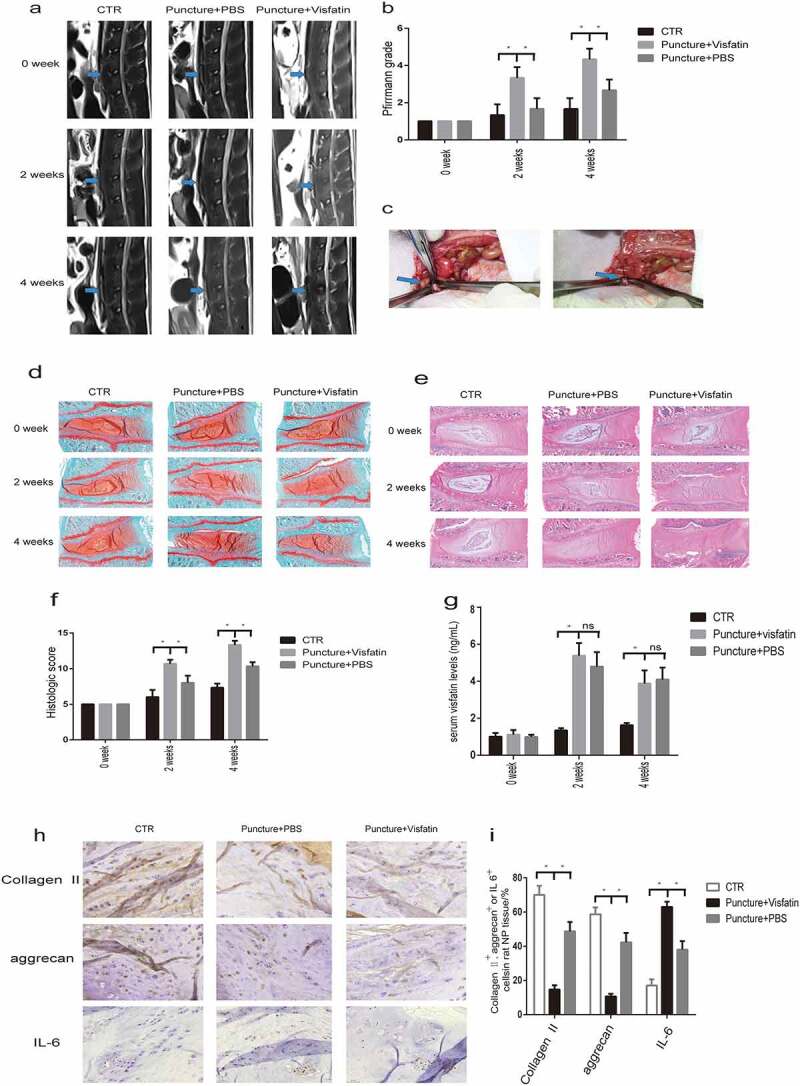
MRI and histological staining showed significantly aggravated disc degeneration in the visfatin group. (a) The intensity of the T2 signal after 2 and 4 weeks of disc puncture was significantly lower in the visfatin group than in the other two groups. (b) The degree of disc degeneration according to Pfirrmann grading system was significantly higher in the visfatin group than in the other two groups. (c) The blue arrow points to the L4/5 IVD that was punctured and injected with visfatin or PBS using a 27-gauge needle. (d, e) Safranin O/fast green and HE staining of the IVDs after 2 and 4 weeks of disc puncture showed more significant IVD disease in the visfatin group. (f) The histological score of the rats in the visfatin group was higher than that of the rats in the other groups after 2 and 4 weeks of disc puncture. (h,i) Immunohistochemistry showed stronger IL-6 immunoreactivity and lower type II collagen and aggrecan expression in the nucleus pulposus cells after 4 weeks of disc puncture in the visfatin group than in the other two groups. (g) ELISA showed a sharply increase of visfatin in the serum 2 and 4 weeks after the puncture. CTR, control. n = 5, *P < 0.001 versus visfatin. The data are represented as the mean ± standard deviation

To investigate the role of visfatin in IVDD in vivo, we established a rat disc degeneration model as described in our previous study (Figure 3c). Magnetic resonance imaging (MRI), H&E staining, and Safranin O/fast green staining data were recorded after disc puncture and injection of visfatin or PBS. MRI showed significantly aggravated disc degeneration in the visfatin group after 2 and 4 weeks of disc puncture compared to that in the other groups (Figure 3a). The results of our MRI-guided quantitative evaluation, which was performed in accordance with the Pfirrmann grading system, confirmed that visfatin has lesion effects on IVD (Figure 3b). Safranin O/fast green and H&E staining results revealed a decrease in disc height, shrinking and even disappearance of the NP in some cases, decrease in the number of NP cells, partial or full replacement of the NP by fibrous tissue, and disorganization of the collagen fibres between 2 and 4 weeks after disc puncture in the visfatin group. In contrast, the PBS group exhibited no significant change in NP between 2 and 4 weeks after disc puncture (Figure 3d and e). The histological score of the rats in the visfatin group was higher than that of the rats in the other two groups (Figure 3f).

IHC staining showed stronger IL-6 immunoreactivity in NP cells after 2 and 4 weeks of disc puncture in the visfatin group compared to those in the other two groups after 4 weeks of disc puncture (Figure 3h and i). However, the expression of type II collagen and aggrecan was lower in the visfatin group (Figure 3h and i). These results indicate that the lesion effects of visfatin on IVD might be associated with type II collagen and aggrecan suppression and IL-6 stimulation.

ELISA results showed that visfatin in the rats plasma inceased sharply in both PBS and visfatin groups two weeks after the puncture. Four weeks after the puncture, visfatin in the rats plasma was still higher in the PBS and visfatin groups than the control group (Figure 3g).

### Visfatin reduces type II collagen and aggrecan expression and promotes MMP3 expression in human NP cells

**Figure 4.**
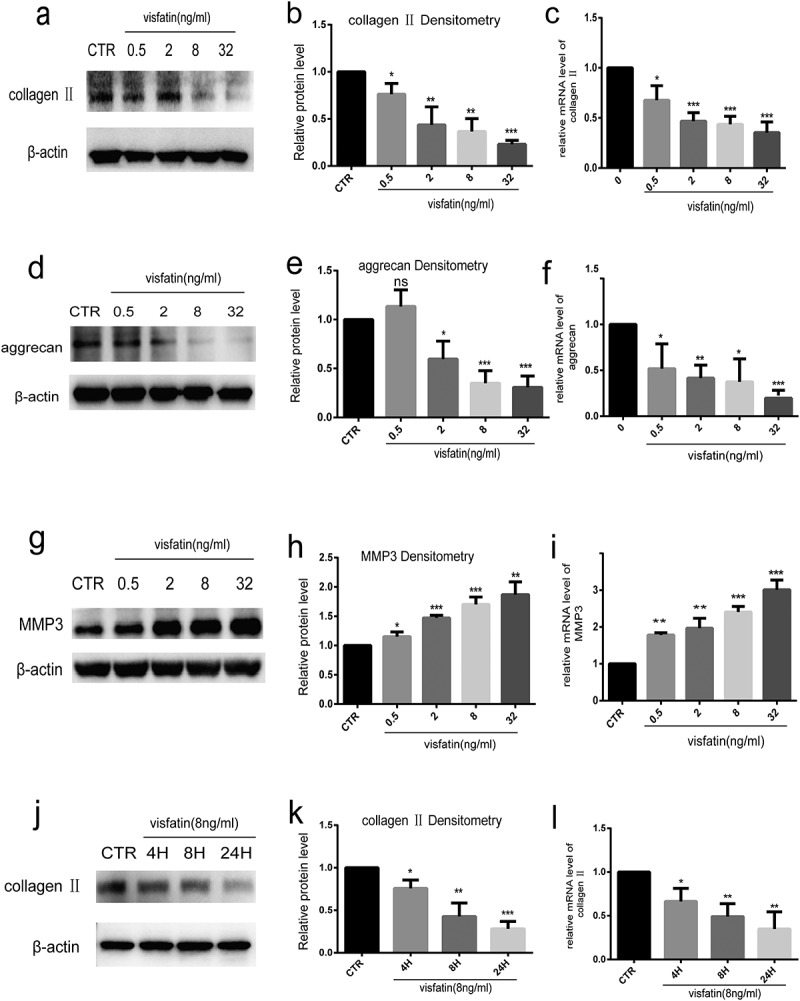
Visfatin reduces type II collagen and aggrecan expression and induces the expression of MMP3 in human NP cells. (a-i) Western blot and RT-qPCR analysis of aggrecan, type II collagen, and MMP3 expressions in NP cells cultured with different doses of visfatin. (j, k, l) Western blot and RT-qPCR analysis of type II collagen level in NP cells treated with visfatin (8 ng/mL) at different time points. CTR, control. n = 3, *P < 0.05, **P < 0.01, ***P < 0.001 versus control. The data are represented as mean ± standard deviation

To investigate the effect of visfatin on NP cells, we treated NP cells with different doses of visfatin (0, 0.5, 2, 8, 32 ng/mL, 24 h). Type II collagen and aggrecan levels decreased in NP cells treated with visfatin in a dose-dependent manner, with the lowest level being observed at 32 ng/mL of visfatin (Figure 4a,b,d and e). RT-qPCR showed that the relative mRNA levels of type II collagen and aggrecan were lowest at 32 ng/mL of visfatin (Figure 4c and f). In contrast, MMP3 level gradually increased in the NP cells treated with visfatin in a dose-dependent manner, with the highest increase being observed at 32 ng/mL of visfatin, compared to the control group (Figure 4g and h). RT-qPCR result for MMP3 was similar to that of western blot analysis (Figure 4i). Analysis of the expression of type II collagen in NP cells exposed to visfatin at different time points (8 ng/mL, 0, 4, 8, and 24 h) with western blot revealed that type II collagen started to decrease at 4 h, with a significant decrease occurring at 8 and 24 h in NP cells (Figure 4j and k). RT-qPCR and western blot analysis results for type II collagen were similar (Figure 4l).

### Visfatin promotes IL-6 expression in human NP cells

**Figure 5.**
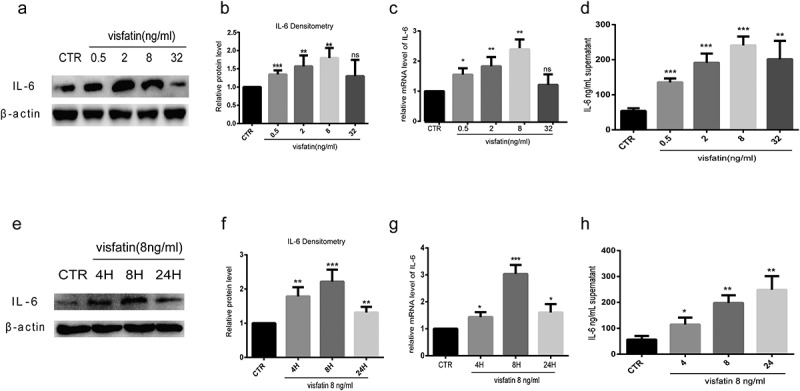
Visfatin induces IL-6 expression in human NP cells. (a-c, e-g) Western blot and RT-PCR analysis of IL-6 expression in NP cells cultured with different doses of visfatin and treated with visfatin (8 ng/mL) at different time points. (d, h) ELISA analysis of IL-6 expression in the supernatant of NP cells cultured with different doses of visfatin and treated with visfatin (8 ng/mL) at different time points. CTR, control. n = 3, *P < 0.05, **P < 0.01, ***P < 0.001 versus control. The data are represented as mean ± standard deviation

To confirm the effect of visfatin on IL-6 expression, NP cells were treated with different doses of visfatin (0, 0.5, 2, 8, 32 ng/mL, 24 h) at the indicated time point. The IL-6 level gradually increased in the NP cells treated visfatin in a dose-dependent manner, with the highest increase being observed at 8 ng/mL of visfatin, compared with the control group (Figure 5a and b). RT-qPCR and ELISA showed similar results (Figure 5c and d). The expression of IL-6 was then detected in NP cells treated with 8 ng/mL visfatin at different time points (0, 4, 8, and 24 h). Western blot analysis and RT-PCR showed that in NP cells treated with visfatin, the expression of IL-6 started to increase at 4 h, with a significant increase occurring at 8 h compared to the control group (Figure 5e,f and g). ELISA results showed the expression of IL-6 started to increase at 4 h, with the highest increase being observed at 24 h (Figure 5h).

### Inhibition of ERK-, JNK-, and p38-mitogen-activated protein kinase (MAPK) signalling pathways block the visfatin-mediated IL-6 expression

**Figure 6.**
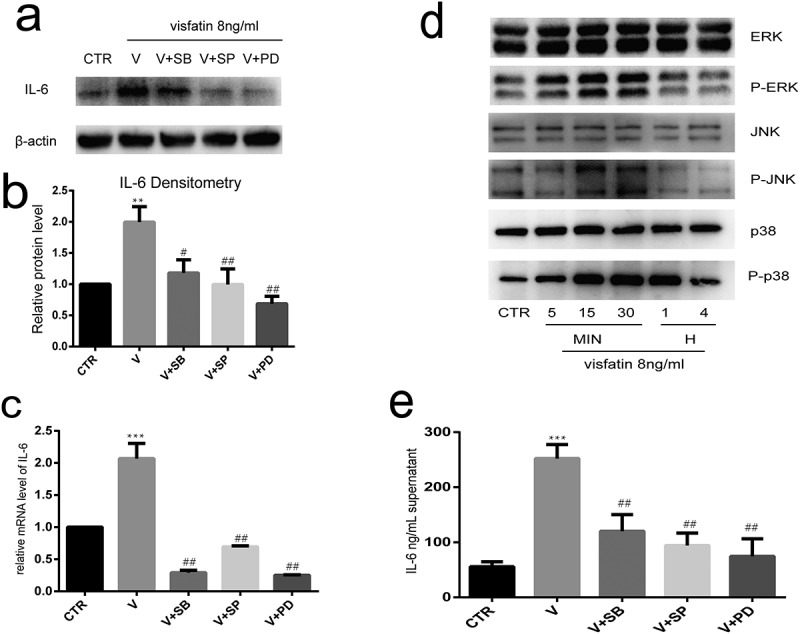
Inhibition of ERK-, JNK-, and p38-MAPK signalling pathways block the visfatin-mediated IL-6 expression. (a, b, c, e) Pretreatment of NP cells with ERK1/2, JNK, and p38 inhibitors significantly inhibited the visfatin-mediated induction of IL-6 mRNA and protein expression. (d) ERK, JNK, and p38 phosphorylation rapidly increased after visfatin treatment. ERK, JNK, and p38 activity levels peaked at 30 min post-treatment with visfatin. CTR, control. V, visfatin. n = 3. **P < 0.01 versus contron. ***P < 0.001 versus control. ^#^P < 0.05 versus visfatin. ^##^P < 0.01 versus visfatin. The data are represented as the mean ± standard deviation

To determine whether visfatin-mediated induction of IL-6 expression requires MAPK signalling, NP cells were pretreated with the indicated ERK1/2 (PD98059), JNK (SP600125), or p38 (SB203580) inhibitors. Pretreatment with the inhibitors significantly impeded the visfatin-mediated induction of IL-6 protein (Figure 6a,b and e) and mRNA (Figure 6c) levels.

To confirm the involvement of ERK-, JNK-, and p38-MAPK signalling pathways in visfatin-mediated induction of IL-6 expression in NP cells, western blot analysis evaluating the activity of ERK-, JNK-, and p38-MAPK signalling pathways was performed with the NP cells treated with visfatin. A rapid increase in ERK, JNK, and p38 phosphorylation was noted after visfatin treatment. ERK, JNK, and p38 activity levels peaked at 30 min post-treatment with visfatin (Figure 6d).

### Specific siRNA-mediated knockdown of p38, ERK, and JNK blocks the induction of IL-6 by visfatin

**Figure 7.**
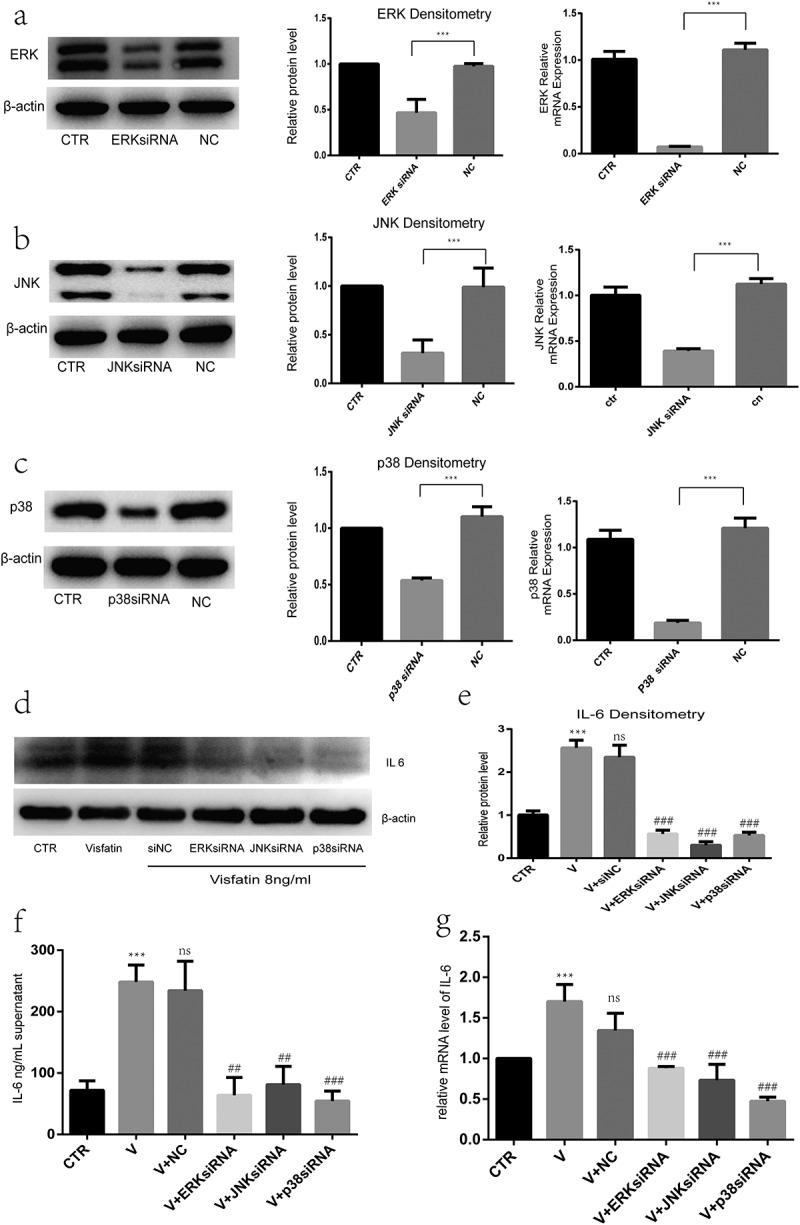
Specific small-interfering RNA (siRNA)-mediated knockdown of ERK, JNK and p38 blocks the induction of IL-6 by visfatin. (a, b, c) ERK, JNK, and p38 siRNAs significantly reduced the ERK, JNK, and p38 mRNA and protein levels, respectively. Nucleus pulposus cells pretreated with ERK, JNK, and p38 siRNAs. (d, e, f, g) Compared to visfatin treatment, pretreatment significantly suppressed the visfatin-mediated induction of IL-6 mRNA and protein levels. CTR, control. V, visfatin. n = 3. ***P < 0.001 versus control. ^##^P < 0.01 versus visfatin. ^###^P < 0.001 versus visfatin.The data are represented as the mean ± standard deviation

To confirm that p38, JNK, and ERK are involved in the visfatin-mediated IL-6 upregulation, specific siRNAs were used to knockdown p38, ERK, and JNK. The ERK, JNK, and p38 siRNAs significantly reduced the mRNA and protein levels (Figure 7a,b and c) of ERK, JNK, and p38, respectively.

Further, to determine whether the ERK-, JNK-, and p38-MAPK signalling pathways are involved in the visfatin-induced IL-6 expression, NP cells were pretreated with ERK, JNK, and p38 siRNAs. Compared to visfatin treatment, pretreatment significantly suppressed the visfatin-mediated induction of IL-6 protein levels (Figure 7d, e and f) and mRNA (Figure 7g).

## Discussion

This study is the first to suggest that visfatin has a stimulatory effect on IVDD development. Our results indicated that visfatin enhances the inflammatory response and aggravates ECM degradation in NP cells. In addition, we also supposed that epidural adipose tissue exerts an inhibitory effect on IVDD development process by reducing the expression of the cytokine, IL-6, and the adipocytokines visfatin.

ECM degradation and reduction in the number of NP cells play an important role in the development of the multifactorial disease, IVDD [32]. Obesity, a known risk factor of IVDD, is now being increasingly recognized as a systemic inflammatory medical condition, and adipocytokines have been identified as potential inflammatory mediators [33]. Recent studies have shown that inflammation might play an essential role in IVDD development [6,7,34]. Leptin, alone or in synergy with TNF-α, IL-1β, or IL-6, significantly increases the expression of proinflammatory cytokines (TNF-α and IL-6) and MMPs [18]. In our previous study, we found that resistin binds to TLR4 and increases the expression of CCL4 in IVD tissues via NF-kB and p38/MAPK activation, which promotes the infiltration of macrophages [14]. However, it is still largely unknown whether other adipocytokines act as mediators.

In our study, we found that the histological characteristics of epidural fat and subcutaneous one were similar. And we showed that the expression of visfatin, leptin, and IL-6 was significantly decreased in epidural adipose tissues. Since IL-6 can induce TNF-α expression and neuronal cell apoptosis in DRG, it may aggravate allodynia and hyperalgesia [12,13]. Recent studies revealed that external pressure could induce IL-6 secretion in brown adipocytes, leading to its inability to decompose fat and regulate the metabolism of glucose and other substances. This results in obesity and its associated complications [35]. Jaclyn et al. found that increased epidural fat results in better physical function among older adults with chronic LBP [36]. The evidence mentioned above supports the theory that epidural fat relieves chronic LBP by reducing the production of IL-6 and hence is protective in nature for the LBP patient subgroup.

NAMPT, also known as visfatin or PBEF, is enhanced in metabolic pathologies and inflammation, events that play an important role in the pathophysiology of musculoskeletal diseases [37]. Recent studies found higher upregulation of visfatin in severe IVDD grades than in mild IVDD grades [18]. However, its direct function in NP cells is yet to be defined.

In this in vivo study, we investigated the role of visfatin in IVDD development. Compared to the non-puncture and PBS groups, significant degenerative changes were observed between 2 and 4 weeks after disc puncture in the visfatin group. While H&E and Safranin O/fast green staining revealed reduced NP cell sizes and disc heights, MRI results showed decreased signal intensities. In the visfatin group, a stronger IL-6 immunoreactivity and lower expression of type II collagen and aggrecan were observed with IHC staining. These findings indicate that visfatin has lesion effects on IVD. We also investigated the visfatin levels in the rats serum two and four weeks after the puncture by ELISA. The results showed a sharply increase two and four weeks after the puncture. We speculated that it was because postoperative stress response and potential infection affected plasma levels of visfarin. In fact, IVD is mainly an avascular tissue; thus, serum visfatin concentration may be poorly related to IVD visfatin levels.

Further, on treating NP cells with different doses of visfatin at indicated time points to confirm the role of visfatin in IVDD, we observed that visfatin reduces type II collagen and aggrecan levels and induces MMP3 production in NP cells. In addition, visfatin also enhanced the expression of IL-6 in NP cells. These results indicate that visfatin can enhance the inflammatory response and aggravate the ECM degradation in NP cells.

Our previous study demonstrated that MAPK plays a critical role in the inflammatory response, which remains strongly associated with disc degeneration [14,38]. In this study, we confirmed that visfatin activates the MAPK signalling pathways. Furthermore, we showed that ERK/JNK/p38-MAPK signalling pathway inhibitors suppress the visfatin-induced IL-6 expression in NP cells. Similarly, silencing studies using siRNAs for ERK, JNK, and p38 showed inhibition of visfatin-induced IL-6 expression. These results highlighted the importance of the ERK/JNK/p38-MAPK signalling pathways in the visfatin-mediated regulation of IL-6 expression. However, the expression of type II collagen and aggrecan remained unchanged upon treatment of NP cells with the MAPK signalling pathway inhibitors or specific siRNAs. We conjectured that visfatin may affect the expression of type II collagen and aggrecan through other signalling pathways or that IL-6 could directly interfere with the expression of type II collagen and aggrecan [39].

There are a few limitations to this study. First, we did not examine the relationship between visfatin and pain development, and the regulatory mechanism underlying this relationship. Second, the exact mechanism underlying the MAPK signalling pathway upstream of IL-6 was not elucidated. Third, the detailed mechanisms of these phenomena are required to be unravelled in further researches. Fourth, the number of patients enrolled in our study was small. Owing to the lack of epidural adipose tissue from healthy individuals, we could only compare the expression of adipocytokines in the epidural adipose and subcutaneous tissues of patients with lumbar spinal stenosis or lumbar disc herniation.

In conclusion, this study provides the first evidence that visfatin reduces the expression of type II collagen and aggrecan and promotes MMP3 expression in NP cells. Furthermore, visfatin induces IL-6 expression in NP cells via the JNK/ERK/p38-MAPK signalling pathways. Thus, epidural fat may have a protective role in inhibiting the development of IVDD and LBP by reducing the production of IL-6. The study results suggested epidural fat and visfatin as potential therapeutic targets for controlling IVDD-associated inflammation.

## Supplementary Material

Supplemental Material

## References

[1] Hartvigsen J, Hancock MJ, Kongsted A, et al. What low back pain is and why we need to pay attention. Lancet. 2018;391(10137):2356–2367. 29573870 10.1016/S0140-6736(18)30480-X

[2] Foster NE, Anema JR, Cherkin D, et al. Prevention and treatment of low back pain: evidence, challenges, and promising directions. Lancet. 2018;391(10137):2368–2383. 29573872 10.1016/S0140-6736(18)30489-6

[3] Roberts S, Evans H, Trivedi J, et al. Histology and pathology of the human intervertebral disc. J Bone Joint Surg Am. 2006;88(suppl):10–14. 10.2106/JBJS.F.00019 16595436

[4] Feng H, Danfelter M, Stromqvist B, et al. Extracellular matrix in disc degeneration. J Bone Joint Surg Am. 2006;88(suppl):25–29. 10.2106/JBJS.E.01341 16595439

[5] Adams MA, Roughley PJ. What is intervertebral disc degeneration, and what causes it? Spine (Phila Pa 1976). 2006;31(18):2151–2161. 16915105 10.1097/01.brs.0000231761.73859.2c

[6] Risbud MV, Shapiro IM. Role of cytokines in intervertebral disc degeneration: pain and disc content. Nat Rev Rheumatol. 2014;10(1):44–56. 24166242 10.1038/nrrheum.2013.160 PMC4151534

[7] Khan AN, Jacobsen HE, Khan J, et al. Chahine. Inflammatory biomarkers of low back pain and disc degeneration: a review. Ann N Y Acad Sci. 2017;1410(1):68–84. 29265416 10.1111/nyas.13551 PMC5744892

[8] Flier JS, Spiegelman BM. Adipogenesis and obesity: rounding out the big picture. Cell. 1996 11 1;87(3):377–389. 8898192 10.1016/s0092-8674(00)81359-8

[9] Francesc V, Rubén C, Joan V, et al. Brown adipose tissue as a secretory organ. Nat Rev Endocrinol. 2017 1;13(1):26–35. 27616452 10.1038/nrendo.2016.136

[10] Pan WW. Martin G Myers Jr. Leptin and the maintenance of elevated body weight. Nat Rev Neurosci. 2018 2;19(2):95–105. 29321684 10.1038/nrn.2017.168

[11] Belluzzi E, Macchi V, Fontanella CG, et al. Infrapatellar fat pad gene expression and protein production in patients with and without osteoarthritis. Int J Mol Sci. 2020 8 21;21(17):6016. 10.3390/ijms21176016 PMC7503946 32825633

[12] Woerden G, Gorter TM, Westenbrink BD, et al. Epicardial fat in heart failure patients with mid-range and preserved ejection fraction. Eur J Heart Fail. 2018 11;20(11):1559–1566. 30070041 10.1002/ejhf.1283 PMC6607508

[13] Zhao C, Liu D, Li H, et al. Dai Expression of leptin and its functional receptor on disc cells: contribution to cell proliferation. Spine (Phila Pa 1976). 2008;33(23):858–864. 10.1097/BRS.0b013e31818338e5 18978578

[14] Segar AH, Fairbank JCT, Urban J. Leptin and the intervertebral disc: a biochemical link exists between obesity, intervertebral disc degeneration and low back pain—an in vitro study in a bovine model. Eur Spine J. 2019;28(2):214–223. 30324498 10.1007/s00586-018-5778-7

[15] Li Z, Shen J, Wu W, et al. Leptin induces cyclin D1 expression and proliferation of human nucleus pulposus cells via JAK/STAT, PI3K/Akt and MEK/ERK pathways. PLoS ONE. 2012;7. 10.1371/journal.pone.0053176 PMC3534060 23300886

[16] Li Z, Wang X, Pan H, et al. Resistin promotes CCL4 expression through toll-like receptor-4 and activation of the p38-MAPK and NF-κB signaling pathways: implications for intervertebral disc degeneration. Osteoarthr Cartil. 2017;25(2):341–350. 10.1016/j.joca.2016.10.002 27737814

[17] Wu M, Tai C, Huang Y, et al. Visfatin promotes IL-6 and TNF-α production in human synovial fibroblasts by repressing miR-199a-5p through ERK, p38 and JNK signaling pathways. Int J Mol Sci. 2018 1 8;19(1):190. 10.3390/ijms19010190 PMC5796139 29316707

[18] Shi C, Wu H, Du D, et al. Nicotinamide phosphoribosyltransferase inhibitor APO866 prevents IL-1β-Induced human nucleus pulposus cell degeneration via autophagy. Cell Physiol Biochem. 2018;49(6):2463–2482. 30261504 10.1159/000493843

[19] Tanaka T, Narazaki M, Kishimoto T. Interleukin (IL-6) Immunotherapy. Cold Spring Harb Perspect Biol. 2018 8 1;10(8):8. 10.1101/cshperspect.a028456 PMC6071487 28778870

[20] Tanaka T, Narazaki M, Kishimoto T. IL-6 in Inflammation, Immunity, and Disease. Cold Spring Harb Perspect Biol. 2014 9 4;6(10):10. 10.1101/cshperspect.a016295 PMC4176007 25190079

[21] Studer RK, Vo N, Sowa G, et al. Human nucleus pulposus cells react to IL-6: independentactions and amplification of response to IL-1 and TNF-α. Spine (Phila Pa 1976). 2011;36(8):593–599. 21178846 10.1097/BRS.0b013e3181da38d5

[22] Patel KP, Sandy JD, Akeda K, et al. Aggrecanases and aggrecanase-generated fragments in the human intervertebral disc at early and advanced stages of disc degeneration. Spine (Phila Pa 1976). 2007;32(23):2596–2603. 17978660 10.1097/BRS.0b013e318158cb85

[23] Murata Y, Rydevik B, Nannmark U, et al. Local application of interleukin-6 to the dorsal root ganglion induces tumor necrosis factor-α in the dorsal root ganglion and results in apoptosis of the dorsal root ganglion cells. Spine (Phila Pa 1976). 2011;36(12):926–932. 21192292 10.1097/BRS.0b013e3181e7f4a9

[24] Murata Y, Nannmark U, Rydevik B, et al. The role of tumor necrosis factoralpha in apoptosis of dorsal root ganglion cells induced by herniated nucleus pulposus in rats. Spine (Phila Pa 1976). 2008;33(2):155–162. 18197099 10.1097/BRS.0b013e3181605518

[25] Noponen-Hietala N, Virtanen I, Karttunen R, et al. Genetic variations in IL6 associate with intervertebral disc disease characterized by sciatica. Pain. 2005;114(1):186–194. 15733644 10.1016/j.pain.2004.12.015

[26] Li Z, Liu H, Yang H, et al. Both expression of cytokines and posterior annulus fibrosus rupture are essential for pain behavior changes induced by degenerative intervertebral disc: an experimental study in rats. J Orthop Res. 2014;32(2):262–272. 24115280 10.1002/jor.22494

[27] Zhang J, Li Z, Chen F, et al. TGF-beta1 suppresses CCL3/4 expression through the ERK signaling pathway and inhibits intervertebral disc degeneration and inflammation-related pain in a rat model. Exp Mol Med. 2017;49(9):e379. 28935976 10.1038/emm.2017.136 PMC5628275

[28] Pfirrmann CW, Metzdorf A, Zanetti M, et al. Magnetic resonance classification of lumbar intervertebral disc degeneration. Spine (Phila Pa 1976). 2001;26(17):1873–1878. 11568697 10.1097/00007632-200109010-00011

[29] Mao HJ, Chen QX, Han B, et al. The effect of injection volume on disc degeneration in a rat tail model. Spine (Phila Pa 1976). 2011 7 15;36(16):E1062–9. 21358491 10.1097/BRS.0b013e3182027d42

[30] Favero M, El-Hadi H, Belluzzi E, et al. Infrapatellar fat pad features in osteoarthritis: a histopathological and molecular study. Rheumatology (Oxford). 2017 10 1;56(10):1784–1793. 28957567 10.1093/rheumatology/kex287

[31] Risbud MV, Guttapalli A, Stokes DG, et al. NP cells express HIF-1 alpha under normoxic culture conditions: a metabolic adaptation to the intervertebral disc microenvironment. J Cell Biochem. 2006;98(1):152–159. 16408279 10.1002/jcb.20765

[32] Ruiz-Fernández C, Francisco V, Pino J, et al. Oreste Gualillo.molecular relationships among obesity, inflammation and intervertebral disc degeneration: are adipokines the common link? Int J Mol Sci. 2019 4 25;20(8):2030. 10.3390/ijms20082030 PMC6515363 31027158

[33] Zhang Y, Liu J, Yao J, et al. Obesity: pathophysiology and intervention. Nutrients. 2014;6(11):5153–5183. 25412152 10.3390/nu6115153 PMC4245585

[34] Vergroesen PP, Kingma I, Emanuel KS, et al. Mechanics and biology in intervertebral disc degeneration: a vicious circle. Osteoarthritis Cartilage. 2015;23(7):1057–1070. 25827971 10.1016/j.joca.2015.03.028

[35] Qing H, Desrouleaux R, Israni-Winger K, et al. Origin and function of Stress-Induced IL-6 in murine models. Cell. 2020 9 17;182(6):1660. 32946784 10.1016/j.cell.2020.08.044 PMC12673928

[36] Sions JM, Rodriguez CA, Pohlig RT, et al. Epidural fat and its association with pain, physical function, and disability among older adults with low back pain and controls. Pain Med. 2018 10 1;19(10):1944–1951. 29024961 10.1093/pm/pnx163 PMC6659021

[37] Francisco V, Pérez T, Pino J, et al. Biomechanics, obesity, and osteoarthritis. The role of adipokines: when the levee breaks. J Orthop Res. 2017;36(2):594–604. 29080354 10.1002/jor.23788

[38] Zhang J, Li Z, Chen F, et al. TGF-b1 suppresses CCL3/4 expression through the ERK signaling pathway and inhibits intervertebral disc degeneration and inflammationrelated pain in a rat model. Exp Mol Med. 2017;49(9):e379. 28935976 10.1038/emm.2017.136 PMC5628275

[39] Studer RK, Vo N, Sowa G, et al. Human nucleus pulposus cells react to IL-6: independent actions and amplification of response to IL-1 and TNF-α. Spine (Phila Pa 1976). 2011 4 15;36(8):593–599. (Phila Pa 1976) 21178846 10.1097/BRS.0b013e3181da38d5

